# Germination ecology of turnip weed (*Rapistrum rugosum* (L.) All.) in the northern regions of Australia

**DOI:** 10.1371/journal.pone.0201023

**Published:** 2018-07-19

**Authors:** Sudheesh Manalil, Hafiz Haider Ali, Bhagirath Singh Chauhan

**Affiliations:** 1 The Centre for Crop Science, Queensland Alliance for Agriculture and Food Innovation (QAAFI), The University of Queensland, Gatton, Queensland, Australia; 2 School of Plant Biology, Institute of Agriculture, The University of Western Australia, Perth, Crawley, Australia; 3 Amrita University, Coimbatore, India; 4 Department of Agronomy, University College of Agriculture, University of Sargodha, Sargodha, Punjab, Pakistan; Brigham Young University, UNITED STATES

## Abstract

In Australia, turnip weed has been rapidly emerging as one of the major weeds in conservation agricultural systems. Germination and emergence of turnip weed were examined for two populations collected from Gatton and St George regions of Australia; two locations with high and low rainfall, respectively. The seeds of turnip weed germinated at all the tested temperatures, but germination was the lowest at 15/5°C, intermediate at 20/10°C and highest at 25/15°C and 30/20°C. The results indicated a high adaptability of turnip weed to warm environmental conditions, although it is a major problem in the winter season. Germination was higher in dark than light/dark regimes except at 30/20°C. Three was a concomitant reduction in germination as the osmotic potential values decreased from 0 to -1.0 MPa. There was 2 and 4% germination at -0.8 MPa for Gatton and St George populations, respectively, and no germination occurred at an osmotic potential of -1.0 MPa. There was a reduction in germination when the sodium chloride (NaCl) concentration was increased from 0 to 150 mM, and no germination was observed at 200 and 250 mM of NaCl. Turnip weed germinated over a broad range of pH (4 to 10). Seedling emergence was higher at 1 cm depth compared to 0.5 cm or at the soil surface. There was 28 and 33% emergence at the surface for the Gatton and St George populations, respectively, compared to 48 and 56% emergence from 1 cm depth for the Gatton and St George populations, respectively and no emergence was observed from 6 cm depth. The results indicated that tillage leading to shallow burial would promote the emergence of turnip weed; on the contrary, tillage that could bury seeds deep into the soil profile might minimise the emergence. Under ideal conditions and lack of integrated weed management programmes, this weed will emerge, set seeds and enrich the soil seed bank and thereby continue to be a problem in the northern grain region of Australia.

## Introduction

Turnip weed (*Rapistrum rugosum* (L.) All.) is a major agricultural weed from the family of Brassicaceae that is rapidly increasing in prevalence in Australia, Iran, USA and Russia [1–4]. In Australia, major patches of turnip weed are observed in wheat (*Triticum aestivum* L.), chickpea (*Cicer arietinum* L.) and other winter crops [5]. Turnip weed is a highly competitive weed; in addition to the cropping areas, this weed is prevalent in the fallow regions, railway tracks, and road side [1, 4, 6]. Weeds of Brassicaceae are rapidly emerging under conservation agricultural systems in Australia as these weeds could adapt to varying environmental conditions and prevailing crop management practices [1,4, 7].

Turnip weed can produce up to 77,000 seeds per plant [8]. Previous reports indicated that this weed could germinate under varying soil and physical environments [6, 9]. When tested in the laboratory environments, turnip weed germinated over a wide range of pH (4 to 10) and there was germination even at a medium level of salinity (160 mM of sodium chloride (NaCl)) [9]. Generally, germination of Brassicaceae weeds and in particular turnip weed is not limited by dark conditions [6, 9, 10]. In addition, the presence of seed coat ensures a physical barrier leading to periodicity in germination and will expose to different environments that are highly conducive for emergence, growth, and reproduction [9].

Turnip weed is a highly competitive weed and around 10 plants m^-2^ could reduce the chickpea yield by 40% [4]. A study conducted in Gatton, Queensland, indicated that a weed density of 18 plants m^-2^ could cause a yield reduction of 50% (Manalil and Chauhan; unpublished data). Being a broadleaf weed, management of this weed is difficult when present in chickpea. Resistance against acetolactate synthase inhibiting herbicides was observed in many Brassicaceae weeds [2, 11, 12]. In Australia, Adkins *et al*. [12] identified chlorsulfuron resistant turnip weed populations way back in the 1990s. In Iran, turnip weed endowing multiple resistance mechanisms against acetolactate synthase inhibiting herbicides were identified [2].

Knowledge on germination ecology of weeds would help to frame the most appropriate weed management options [13, 14]. Exposure of weed seeds to varying environments and prevailing agronomic management would significantly affect the germination and emergence of weeds [15–17]. Drought conditions, inherent salinity, and soil pH may affect weed germination and emergence differentially [14–16]. Chauhan *et al*. [9] explored the germination biology of turnip weed in a South Australian population. However, those results may not be fully applicable to turnip weed populations of Queensland owing to the difference in weather, soil type and crops. Mediterranean type weather with rainy winter and dry summer prevails in South Australia [18]. On the contrary, the weather is quite varying across Queensland with hot humid summer (wet season) and mild to warm winter. In addition, in Queensland, considerable variation exists in day time temperature even during peak winter time [18]. Environmental conditions during the seed development may affect the germination characteristics of plants [19], as there would be differential supply of nutrients and hormones to developing embryo with varying growth environments [19, 20]. In Queensland, considerable variation exists in terms of cumulative rainfall and its distribution between locations [18], therefore, it is likely that weed populations vary in their response to biotic and abiotic factors. With all these backgrounds, a study was conducted to examine the effect of light, temperature, salt, osmotic stress, pH, and burial depth of weed seeds on germination and emergence of two populations of turnip weed collected from two locations in Queensland with contrasting rainfall patterns.

## Materials and methods

### Seed description and details of sites

Seeds were collected from St George (RRS) and Gatton (RRG) in November 2015, low (500 mm) and high rainfall (770 mm) areas in Queensland, respectively. St George is 325 km (aerial distance) away from Gatton and at an elevation of 200 m above mean sea level (AMSL); whereas, elevation of Gatton is 89 m (AMSL) [18]. Soil of St George is redsodosl with bulk density and pH of 1.40 g cm^-3^ and 8.4, respectively; soil of Gatton is Grey Vertosol with a bulk density and pH of 1.32 g cm^-3^ and 7.2, respectively [21]. Although difference in mean annual rainfall is only around 200 mm, Gatton receives well distributed and assured summer rains compared to St George [18]. In 2015, Gatton received an annual rainfall of 681 mm in 99 rainy days and St George received a total rainfall of 426 mm in 66 rainy days. In 2014–15 period, Gatton received 341 mm of summer rainfall compared to 90 mm of rainfall for St George. Although both the seed collection sites practice conservation tillage and totally depend on rainfall, cropping in these sites differs as only winter crop is raised at St George (chickpea or wheat), whereas, both winter (chickpea or wheat) and summer (sorghum (*Sorghum bicolor* (L.) Moench)) crops are raised in Gatton due to adequate summer showers. The RRS population was collected from a chickpea field (S28°11.104’, E 148° 38.054’) and RRG populations from a wheat field (S27° 33.552’, E 152° 19.443’). Fully matured seeds (from plants that were completely senesced) were collected by gently tapping the inflorescence into a basin. Populations were collected from around 50 plants distributed in an area of around 5 ha. Collected seeds were kept in paper bags and stored in a fully ventilated rain out facility at the Gatton research facility of the University of Queensland until used in the experiments (May to August 2016).

### Experiments on temperature and light

Naked seeds were used to examine the effect of temperature and light as there was no germination of seeds with silique intact. The assessment was carried out by placing 30 seeds evenly in a 9 cm diameter Petri dish with two Whatman No.1 filter papers and moistened with 5 ml of distilled water. Petri dishes were covered with zip lock plastic bags to minimise moisture loss and placed in an incubator set at day/night alternating temperature (15/5, 20/10, 25/15 and 30/20°C) with photoperiod coinciding high temperature. Germination was assessed both under the light and dark regimes after three weeks. Darkness was simulated by covering Petri dishes with two layers of aluminium foil immediately after placing seeds. Initial germination was continued up to 3 weeks and visible protrusion of radicle was counted at a weekly interval.

### Effect of osmotic stress, salt stress and pH on germination

The effect of water stress was assessed by preparing solutions with osmotic potential 0.0, -1, -0.2, -0.4, -0.6,-0.8 and -1.0 MPa by dissolving 0.0, 93.6, 132.4, 187.2, 229.2, 264.7 and 295.9 g of polyethylene glycol 8000 in 1 L of distilled water, respectively [22]. Germination was assessed under sodium chloride (NaCl) stress, osmotic stress and pH at the day/night alternating temperature of 25/15°C as there was the highest germination at this temperature regime in the temperature and light experiment. The effect of salt stress was studied by NaCl solutions of 0, 25, 50, 100, 150, 200 and 250 mM. To examine the effect of pH, buffer solutions were prepared by following the procedures of Chauhan *et al*. [9]. A 2-mM solution of MES [2-(*N*-morpholino) ethanesulfonic acid] was adjusted to pH 5 or 6 with 1 N hydrogen chloride (HCl) or sodium hydroxide (NaOH). A 2-mM solution of HEPES [*N*-(2-hydroxymethyl) piperazine-*N*-(2-ethanesulfonic acid)] was adjusted to pH 7 or 8 with 1 N NaOH. A pH 9 or 10 buffer was prepared with 2-mM tricine [N-Tris (hydroxymethyl) methylglycine] and adjusted with 1 N NaOH. Unbuffered deionized water (pH 6.7) was used as a control.

### Effect of burial depth on emergence

The effect of seed burial depth was studied by placing 30 seeds at 0, 1, 2, 3, 4 and 6 cm depths. The soil used in this experiment was collected from the Gatton Research Farm of the University of Queensland. The soil of the experimental site had a pH of 7.2, organic matter of 2.7%, nitrogen of 33 mg kg^-1^, phosphorus of 215 mg kg^-1^ and potash of 412 mg kg^-1^. The soil was filtered through a 4 mm sieve and then filled in pots of 12 cm diameter (four replications) and maintained under the rainout shelter facility at the University of Queensland under an irrigated environment.

### Statistical analyses

A randomised complete block design was used in all the experiments with three replicates for Petri dish assays and four replicates for the pot studies. In the laboratory study, blocking was done by placing Petri dishes on different shelves of the incubator and in the pot study by grouping the pots of the same replicate together. All experiments were repeated twice. Data were pooled for the analysis as there was no time by treatment interaction. Analysis of variance was performed on the data from the light and temperature experiment, effect of pH and burial depth experiments. Non-linear regression analysis was performed on osmotic potential and salinity experiments. Germination percentage was fitted to a functional three parameter sigmoid model using Sigmaplot software. The model fitted was

$$G\;(\%)={Gmax/(1+exp}^{\left(-(x-x0)/b\right)})$$ (1)

where *G* (%) is the percentage of germination, *G*_*max*_ is the maximum germination as per the fitted model, *x* is treatment level or concentration, *x0* is the treatment level or concentration corresponds to 50% germination or emergence, and *b* is the slope [23].

## Results

### Effect of temperature and light on germination

**Fig 1:**
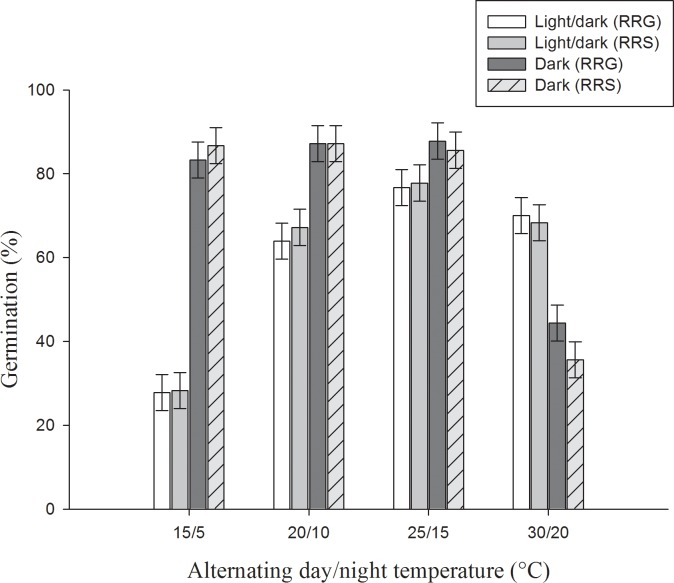
Effect of alternating day/night temperatures and light regimes on seed germination of turnip weed seeds from Gatton (RRG) and St George (RRS) incubated at 15/5, 20/10, 25/15 and 30/20°C light/dark and dark in a 12-h photo-period for 21 days. Error bars are LSD (p≤0.05, n = 6).

No germination was observed when freshly harvested seeds were tested with silique intact (data not shown); however, germination improved significantly when naked seeds were used in the experiment (Fig 1). When tested at varying temperature regimes, germination of turnip weed was affected by the tested temperature and light, although population difference was not observed except at 30/20°C for the dark treatment (Fig 1). Germination was less than 29% at 15/5°C day/night temperature for RRG and RRS populations. At 20/10°C, germination was higher than 15/5°C but was lower than 25/15 and 30/20°C. Germination was more than 85% for both the populations under the dark environment in all the temperature ranges except for 30/20°C where germination was less than 45%, indicating sensitivity to darkness at high temperature. In addition, unlike other temperature regimes, difference between populations was observed for their response to dark at 30/20°C.

### Effect of osmotic stress, salinity and pH on germination

**Fig 2:**
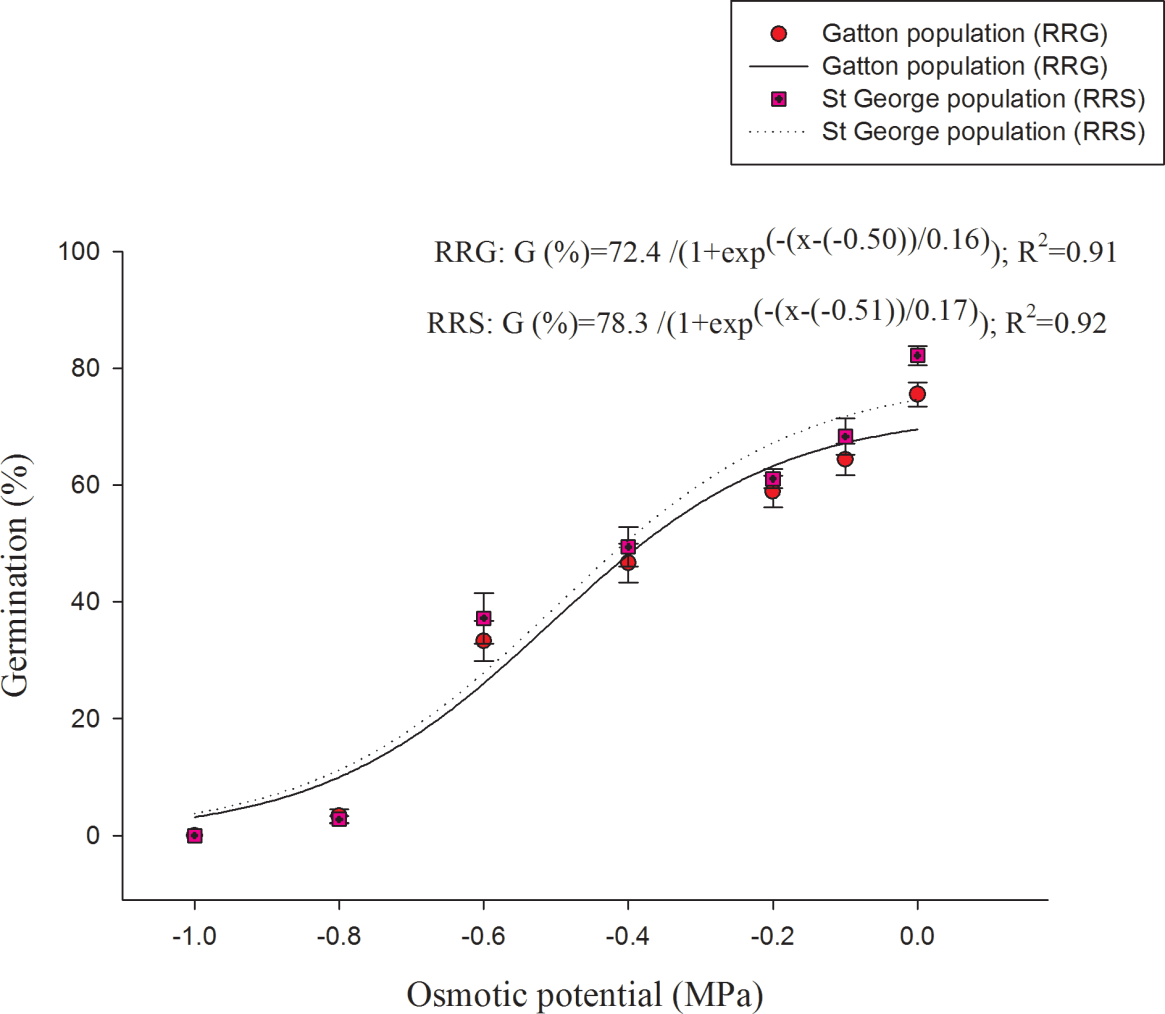
Effect of osmotic potential on the germination of two populations of turnip weed from St George (RRS) and Gatton (RRG) incubated at 25/15°C day/night temperatures in a 12-h photoperiod for 21 days. Lines represent the functional three- parameter sigmoid model fitted to the data. Error bars are standard error of mean (n = 6).

A three-parameter sigmoid model fitted to the germination data (%) corresponds to the osmotic potential values (Fig 2). There was a concomitant reduction in germination as osmotic potential values decreased from 0 to -1.0 MPa. Germination was only 2 and 4% at -0.8 MPa for RRG and RRS populations, respectively, and no germination was recorded at -1.0 MPa. Osmotic potentials that can cause 50% reduction in germination based on the regression models were -0.50 and -0.51 MPa, for RRG and RRS populations, respectively.

**Fig 3:**
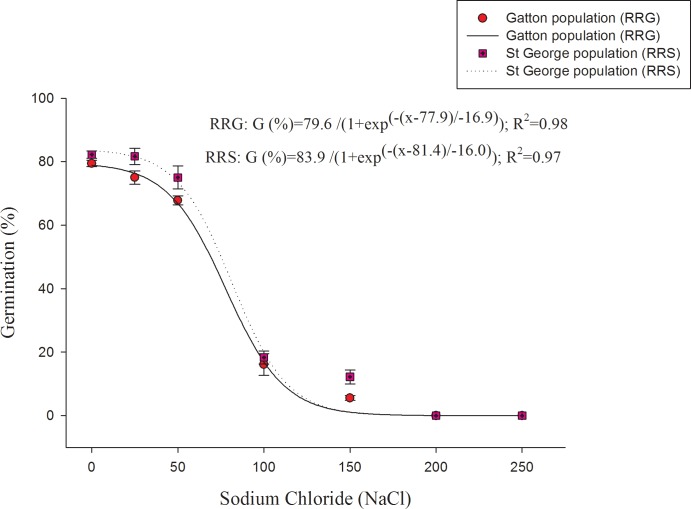
Effect of sodium chloride (NaCl) on the emergence of two populations of turnip weed from St George (RRS) and Gatton (RRG) incubated at 25/15°C day/night temperatures in a 12-h photoperiod for 21 days. Lines represent the functional three-parameter sigmoid model fitted to the data. Error bars are standard error of mean (n = 6).

**Fig 4:**
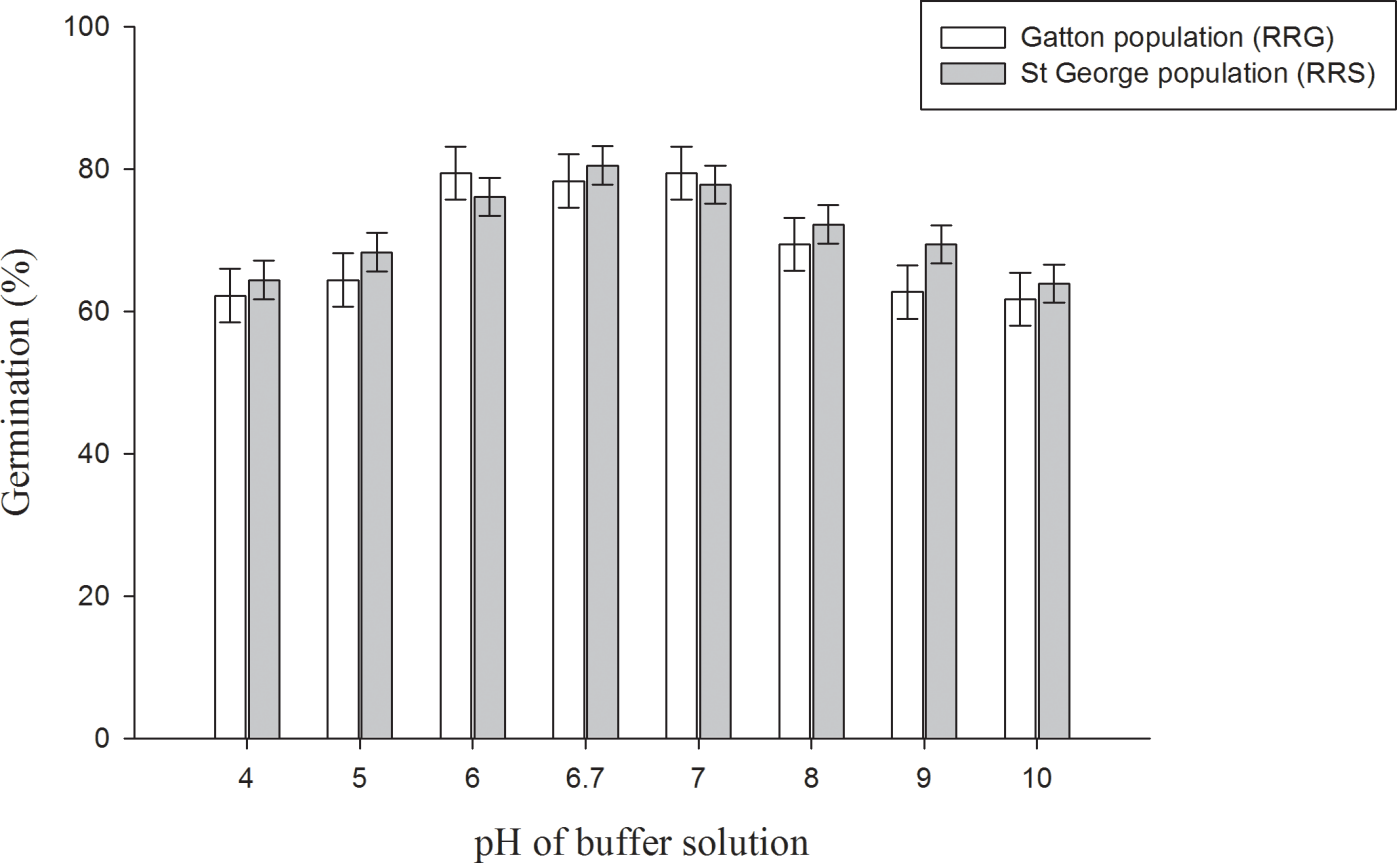
Effect of buffered pH solutions on the germination of two populations of turnip weed from St George (RRS) and Gatton (RRG) incubated at 25/15°C day/night temperatures in a 12-h photoperiod for 21 days. Error bars are LSD (p≤0.05, n = 6).

A three-parameter sigmoid model was fitted to the germination data obtained at different concentrations of NaCl (Fig 3). There was a reduction in germination when NaCl concentration was increased from 0 to 150 mM (Fig 3) and no germination was observed beyond this concentration. Germination was 6 and 12% at 150 mM for RRG and RRS populations, respectively. The concentration for 50% inhibition of the maximum germination, estimated from the fitted model, was 77 and 81 mM NaCl for the RRG and RRS populations, respectively (Fig 3). Turnip weed could germinate over a broad range of pH. Turnip weed germination was more than 75% over a pH range of 6 to 7. However, a reduction in germination was observed at pH lower than 6 and higher than 7 (Fig 4).

### Effect of seed burial depth on emergence

**Fig 5:**
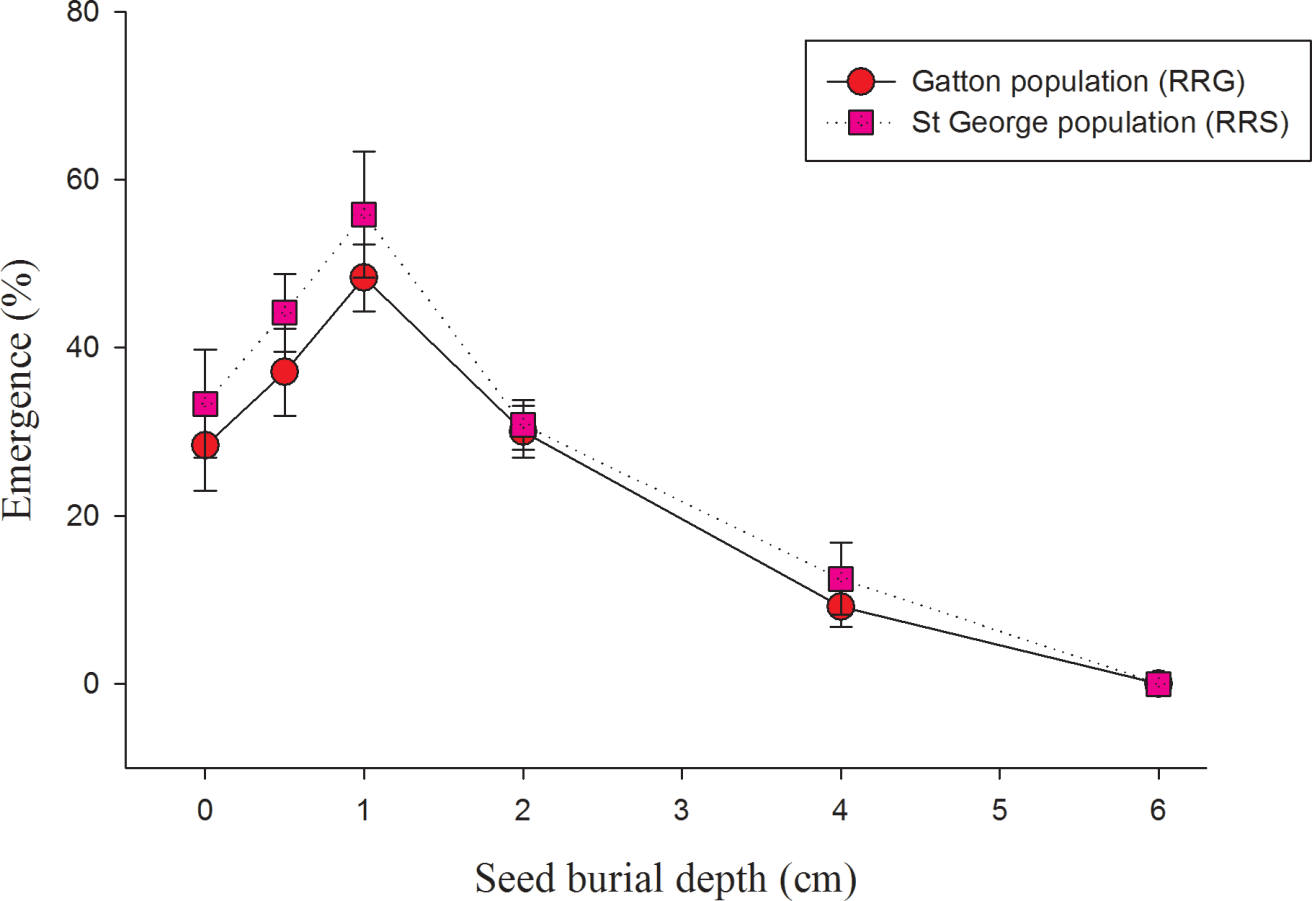
Effect of burial depth on seedling emergence of two populations of turnip weed from St George (RRS) and Gatton (RRG) in a pot study for 21 days. Error bars are standard error of mean (n = 8).

The seed burial depth experiment indicated that the emergence was lower at the soil surface compared to 0.5 cm and 1 cm depths (Fig 5). There was 28 and 33% emergence at the surface for the RRG and RRS populations, respectively, compared to 48 and 56% emergence at 1 cm depth for the RRG and RRS populations, respectively. Emergence was only 9 and 13% at 4 cm depths for the RRG and RRS populations, respectively, and no germination was observed at 6 cm depth.

## Discussion

The results of this study vary from that carried out in South Australia where germination was not affected by the varying temperature regimes under the light environment [9]; however, in this study, germination was higher under warmer environmental conditions than cooler temperature conditions, although it is a major problem weed in the winter season. High germination in complete darkness was observed in this study. Photo inhibition in turnip weed was observed in an earlier study carried out in New South Wales in the 1990s [6]. There was enhanced germination in turnip weed populations studied in Iran under dark conditions when naked seeds were stored (prior to experiment) at constant temperature of 3 and 25°C [24]. However, in the current study, seeds were stored under ambient conditions and high germination in darkness was observed. However, in the study carried out in South Australia, germination was improved by exposure to light over complete darkness [9]. Annual ground cherry (*Physalis divaricata* L.) populations from Iran exhibited variation in seed dormancy and this was related to the temperature conditions at seed maturity; seeds developed at warmer temperature exhibited less dormancy compared to cooler temperature [25]. In a study, adjacent populations of ripgut brome (*Bromus diandrus Roth*.) from crop field and fence line exhibited a variation in dormancy characteristics, difference in crop management practices were hypothesised to be the reason behind this difference [26]. This difference between populations from South Australia and Queensland in their germination response to dark could be ascribed to the difference in weather, difference in cropping systems [5], and changes that have been occurring in the agronomic management over time. In South Australia, Mediterranean weather prevails with predominant cropping in the winter season; contrary to warmer subtropical or tropical weather in Queensland with both summer and winter dominant cropping systems.

Other researchers have reported that seed germination of Brassicaceae weeds could be affected by light and temperature conditions. For example, germination of African mustard (*Brassica tournefortii* Gouan.) was significantly inhibited by the lower temperature (15/9°C) compared to higher temperature [10]. In another study, Kleemann *et al*. [27] observed a reduced germination of perennial wall rocket (*Diplotaxis tenuifolia* (L.) DC.) at lower temperatures (10 to 20°C) compared to higher temperature. Germination of Oriental mustard (*Sisymbrium orientale* L.) was significantly higher at 25/15°C compared to low temperatures (15/5 or 20/10°C) [28]. Germination of naked seeds was greater than the seed in intact silique. Under field conditions seeds release dormancy rapidly and substantial seeds germinated in field within a period of three months (Manalil and Chauhan; unpublished data). However, intact seed coat allows this weed to extend the periodicity in germination as dormancy (due to seed coat) release will not be abrupt making the management difficult [9]. In a nut shell, the ability to germinate under varying temperature conditions, darkness and dormancy of freshly harvested seeds favour turnip weed to adapt to diversified environments.

The osmotic potential study indicated that turnip weed has adaptability to water stress environments. The results are in agreement with the earlier observations of Chauhan *et al*. [9] and illustrates the prevalence of this weed in roadsides, railway tracks and fallow areas [1, 6]. A substantial portion of soils in the northern regions of Australia is vertosol where surface layers dry rapidly [29]; however, turnip weed may emerge under water-limiting environments.

Turnip weed exhibited a moderate level of tolerance to different salinity levels (NaCl concentrations) (Fig 3). The present study is in agreement with the earlier study carried out on a South Australian populations of turnip weed [9]. The results are important as salinity is a major production constraint of Australian soils and can limit plant growth [30]. Another Brassicaceae weed, African mustard had shown some level of salt tolerance when tested under laboratory environment [10]. The results of the pH experiment indicated that pH might not be a limiting factor for the germination and emergence of turnip weed.*—*Similarly, there was more than 45% germination of musk weed (*Myagrum perfoliatum* L.) over a pH range of 4–10 [31]. Seeds of African mustard germinated over a broad range of pH from 4–10 [10]. The response of turnip weed to salinity and pH indicates that the weed can thrive extreme soil conditions. The results are important as salinity and alkalinity are often associated and is a major production constraint of Australian soils [32]; however, turnip weed could cope with extreme levels of salinity and pH and continue to spread even under conditions that limit crop production.

The results of burial depth study indicate that shallow burial could increase the emergence of turnip weed (Fig 5). This may be due to better soil, moisture, and seed contact and darkness may not limit germination [9]. Seed germination decreased substantially with increasing soil depths and this pattern is observed in many weeds [33–36]. The results indicated that conducting occasional shallow tillage may not reduce the emergence of turnip weed; on the contrary, deep inversion tillage may reduce the weed emergence. Tillage is a recommended option to manage heavy weed infestations [37]. Different burial depths examined in this study has relevance as crops like cotton (*Gossypium hirsutum* L.) requires intensive tillage and this crop could be rotated with cereal crops [38]. In this study, seeds were unable to emerge from deeper soil layers indicating inversion tillage can be a weed management strategy under heavy infestation. Small seeded weeds like turnip weed fails to emerge as the carbohydrate reserve may not support the seedling growth through the soil profile [14, 39]. Results are in agreement with the similar studies carried out in other broadleaf weeds [14, 39].

Generally, environmental conditions and water availability during seed maturity (maternal environments) would strongly influence the germination and dormancy rates [40]. Contrasting rainfall patterns exist in St George and Gatton locations and variations in emergence pattern between the populations were expected. Considerable variation exists between sites in terms of soil properties, total amount of rainfall and its distribution. Field visits in different parts of the region and discussion with agronomists indicated that a proportion of turnip weed could germinate during summer season producing seeds depending on rainfall. Gatton offers more favourable environment for turnip weed to establish in summer as the region receives more summer rainfall and variability between populations were expected. However, germination characteristics were not inferior for population sourced from the low rainfall area (St George) compared to the high rainfall area (Gatton). Overall, turnip weed is highly adapted to agricultural areas of Queensland where a considerable variation in weather, soil and crop management exists. The biological potential and ecological adaptability of turnip weed to conservation agricultural systems would favour this weed to increase in prevalence under the current agronomic and weed management system.

## Conclusion

Potential of turnip weed to germinate under varying temperature and light regimes point to the adaptability of this weed to infest the cropping regions and fallows of the northern regions of Australia. The results show the potential of turnip weed to thrive occasional water stress and cope up with the inherent soil variability due to salinity, soil pH, and soil moisture retention. Management options should target summer, winter and fallow phase of cropping seasons as turnip weed has the potential to emerge under diversified environments and enrich soil seed bank. The results of the seed burial study indicated that tillage leading to shallow burial would promote the emergence of turnip weed; on the contrary, tillage that could bury seeds deep into the soil profile might minimise the emergence. This indicates the potential of soil inversion tillage to reduce the weed infestation level. The results indicted a high adaptability of turnip weed to the prevailing agronomic management under the conservation systems. Under ideal conditions and lack of integrated weed management programmes, this weed may continue to be a problem in the northern grain regions of Australia.

## Supporting information

S1 File Data set used in the analysis. (XLSX)

## References

[1] Osten VA, Walker SR, Storrie A, Widderick M, Moylan P, Robinson GR, et al Survey of weed flora and management relative to cropping practices in the north-eastern grain region of Australia. Aust J Exp Agr. 2007;47:57–70. 10.1071/ea05141

[2] Hatami ZM, Gherekhloo J, Rojano-Delgado AM, Osuna MD, Alcantara R, Fernandez P, et al Multiple mechanisms increase levels of resistance in *Rapistrum rugosum* to ALS herbicides. Front Plant Sci. 2016;7 10.3389/fpls.2016.00169 26941749 PMC4761845

[3] Simmons MT. Bullying the bullies: The selective control of an exotic, invasive annual (*Rapistrum rugosum*) by oversowing with a competitive native species (*Gaillardia pulchella*). Restor Ecol. 2005; 13:609–15. 10.1111/j.1526-100X.2005.00078.x

[4] Whish JPM, Sindel BM, Jessop RS, Felton WL. The effect of row spacing and weed density on yield loss of chickpea. Aust J Agr Res. 2002; 53:1335–40. 10.1071/ar01168

[5] GRDC. Growing regions, Grains Research Development Corporation, Australia. 2017; https://grdc.com.au/about/our-industry/growing-regions. Accessed Dec 2017.

[6] Cousens R, Armas G, Baweja R. Germination of *Rapistrum rugosum* (L) ALL from New-South-Wales, Australia. Weed Res. 1994; 34:127–35. 10.1111/j.1365-3180.1994.tb01980.x

[7] Chauhan BS, Gill G, Preston C. Influence of environmental factors on seed germination and seedling emergence of Oriental mustard (*Sisymbrium orientale*). Weed Sci. 2006; 54:1025–31. 10.1614/ws-06-092.1

[8] Wilson BJ, Wilson JT. Effect of time of seedling emergence on seed production and time to flowering of eight weeds, 6th Australian Weeds Conference.' Gold Coast, Queensland, Australia, 1981. (Queensland Weed Society. Available at http://wwwcawsorgau/awc/1981/awc198110351pdf.

[9] Chauhan BS, Gill G, Preston C. Factors affecting turnipweed (*Rapistrum rugosum*) seed germination in southern Australia. Weed Sci. 2006; 54:1032–6. 10.1614/ws-06-060r1.1

[10] Chauhan BS, Gill G, Preston C. African mustard (*Brassica tournefortii*) germination in southern Australia. Weed Sci. 2006; 54:891–7. 10.1614/ws-06-053r.1

[11] Boutsalis P, Powles SB. Seedbank characteristics of herbicide-resistant and susceptible *Sisymbrium orientale*. Weed Res. 1998; 38:389–95.

[12] Adkins SW, Wills D, Boersma M, Walker SR, Robinson G, McLeod RJ, et al Weeds resistant to chlorsulfuron and atrazine from the north-east grain region of Australia. Weed Res. 1997; 37:343–9. 10.1046/j.1365-3180.1997.d01-56.x

[13] Widderick M, Walker S, B S. Better management of Sonchus oleraceus L. (common sowthistle) based on the weed's ecology. Pages 535–537 in Sindel BM, Johnson SB, eds 14th Australian Weeds Conference Wagga Wagga, NSW, Australia: Weed Society of New South Wales. 2004.

[14] Chauhan BS, Gill G, Preston C. Factors affecting seed germination of annual sowthistle (*Sonchus oleraceus*) in southern Australia. Weed Sci. 2006; 54:854–60. 10.1614/ws-06-047r.1

[15] Ahmed S, Opena JL, Chauhan BS. Seed germination ecology of doveweed (*Murdannia nudiflora*) and its implication for management in dry-seeded rice. Weed Sci. 2015; 63:491–501. 10.1614/ws-d-14-00115.1

[16] Opena JL, Chauhan BS, Baltazar AM. Seed germination ecology of *Echinochloa glabrescens* and Its implication for management in rice (*Oryza sativa* L.). Plos One. 2014; 9 10.1371/journal.pone.0092261 24642568 PMC3958481

[17] Ramesh K. Weed problems, ecology, and management options in conservation agriculture: issues and perspectives. Adv Agron.131:251–303.

[18] Climate statistics for Australian locations. Bureau of Meteorology, Australian Government, Canberra, ACT. Available at: www.bom.gov.au. Accessed Dec. 2017.

[19] Roach DA, Wulff RD. Maternal effects in plants. Annu Rev Ecol Syst. 1987;18:209–35. 10.1146/annurev.ecolsys.18.1.209

[20] Gorecki MJ, Long RL, Flematti GR, Stevens JC. Parental environment changes the dormancy state and karrikinolide response of *Brassica tournefortii* seeds. Ann Bot. 2012; 109:1369–78. 10.1093/aob/mcs067 22492259 PMC3359922

[21] ASRIS. National soil grids. Australian Soil Resource Information System, Canberra, ACT. Available at: www.asris.csiro.au/mapping/viewer.htm. 2017.

[22] Michel BE. Evaluation of the water potentials of solutions of polyethylene glycol 8000 both in the absence and presence of other solutes. Plant Physiol. 1983; 72:66–70. 16662983 10.1104/pp.72.1.66 PMC1066170

[23] R Development Core Team. R: a language and environment for statistical computing. R foundation for statistical computing. http://www.R-project.org. Accessed Dec. 2017.

[24] Ohadi S, Mashhadi HR, Tavakol-Afshari R. Effects of storage and burial on germination responses of encapsulated and naked seeds of turnipweed (*Rapistrum rugosum*) to light. Weed Sci. 2011;59:483–8. 10.1614/WS-D-10-00153.1

[25] Nosratti I, Heidari H, Muhammadi G, Saeidi M. Germination and emergence characteristics of annual ground cherry (*Physalis divaricata*). Jordan J Biol Sci. 2016;9:131–8.

[26] Kleemann SGL, Gill GS. Seed dormancy and seedling emergence in ripgut brome (Bromus diandrus) populations in Southern Australia. Weed Sci. 2013;61:222–9. 10.1614/WS-D-12-00083.1

[27] Kleemann SGL, Chauhan BS, Gill GS. Factors affecting seed germination of perennial wall rocket (*Diplotaxis tenuifolia*) in southern Australia. Weed Sci. 2007; 55:481–5. 10.1614/ws-06-197.1

[28] Karimmojeny H, Rezvani M, Zaefarian F, Nikneshan P. Environmental and maternal factors affecting on oriental mustard (*Sisymbrium orientale* L.) and musk weed (*Myagrum perfoliatum* L.) seed germination. Braz J Bot. 2014; 37:121–7. 10.1007/s40415-014-0058-1

[29] Dalal RC, Weston EJ, Strong WM, Probert ME, Lehane KJ, Cooper JE, et al Sustaining productivity of a vertosol at Warra, Queensland, with fertilisers, no-tillage or legumes. Effect of duration of lucerne ley on soil nitrogen and water, wheat yield and protein. Aust J Exp Agr. 2004; 44:1013–24. 10.1071/ea03166

[30] Dang YP, Routley R, McDonald M, Dalal RC, Singh DK, Orange D, et al Subsoil constraints in Vertosols: Crop water use, nutrient concentration, and grain yields of bread wheat, durum wheat, barley, chickpea, and canola. Aust J Agr Res. 2006;57:983–98. 10.1071/AR05268

[31] Honarmand SJ, Nosratti I, Nazari K, Heidari H. Factors affecting the seed germination and seedling emergence of muskweed (*Myagrum perfoliatum*). Weed Biol Manag. 2016; 16:186–93. 10.1111/wbm.12110

[32] Dang YP, Christopher JT, Dalal RC. Genetic diversity in barley and wheat for tolerance to soil constraints. Agronomy. 2016; 6 10.3390/agronomy6040055

[33] Chauhan BS, Johnson DE. Seed germination and seedling emergence of synedrella (*Synedrella nodiflora*) in a tropical environment. Weed Sci. 2009; 57:36–42. 10.1614/ws-08-015.1

[34] Chauhan BS, Johnson DE. Germination ecology of two troublesome asteraceae species of rainfed rice: Siam weed (*Chromolaena odorata*) and coat buttons (*Tridax procumbens*). Weed Sci. 2008; 56:567–73. 10.1614/ws-07.200.1

[35] Nandula VK, Eubank TW, Poston DH, Koger CH, Reddy KN. Factors affecting germination of horseweed (*Conyza canadensis*). Weed Sci. 2006; 54:898–902. 10.1614/WS-06-006R2.1

[36] Lu P, Sang WG, Ma KP. Effects of environmental factors on germination and emergence of Crofton weed (*Eupatorium adenophorum*). Weed Sci. 2006; 54:452–7. 10.1614/ws-05-174r1.1

[37] Dang YP, Seymour NP, Walker SR, Bell MJ, Freebairn DM. Strategic tillage in no-till farming systems in Australia's northern grains-growing regions: I. Drivers and implementation. Soil Till. Res. 2015;152:104–14. 10.1016/j.still.2015.03.009

[38] CottonInfo. Stewardship: Reducing the risk of resistance. CottonInfo, Narrabri, NSW. Available at: http://cottoninfo.com.au/stewardship. 2016.

[39] Nosratti I, Abbasi R, Bagheri A, Bromandan P. Seed germination and seedling emergence of Iberian starthistle (*Centaurea iberica*). Weed Biol Manag. 2017;17:144–9. 10.1111/wbm.12128

[40] Eslami SV, Gill GS, McDonald G. Effect of water stress during seed development on morphometric characteristics and dormancy of wild radish (*Raphanus raphanistrum* L.) seeds. Int J Plant Prod. 2010; 4:159–68.

